# Achilles Tendon Shear Wave Velocity Within a 1‐Year Follow‐Up After Non‐Operatively Treated Rupture

**DOI:** 10.1002/jor.70201

**Published:** 2026-04-01

**Authors:** Maria Sukanen, Ra'ad M. Khair, Iida Laatikainen‐Raussi, Aleksi Reito, Ville Ponkilainen, Neil J. Cronin, Arto J. Hautala, Taija Finni

**Affiliations:** ^1^ Faculty of Sport and Health Sciences University of Jyväskylä Jyväskylä Finland; ^2^ Department of Orthopaedics Tampere University Hospital Tampere Finland; ^3^ School of Sport & Exercise University of Gloucestershire Gloucestershire UK

**Keywords:** daily step count, diagnostic imaging, elastography, mechanical properties, muscle strength, patient function, tendon healing, ultrasound

## Abstract

The purpose of this study was to investigate changes in Achilles tendon (AT) shear wave velocity (SWV) during the first year after rupture. Additionally, we aimed to explore relationships between AT SWV and plantarflexion maximal voluntary torque (MVT), steps/day, patient‐reported function, and tendon thickness. Participants (*N* = 27, 8 females, 41.5 ± 9.8 years) were measured for AT SWV at rest, accelerometer‐monitored daily steps, and tendon thickness at 2‐, 6‐, and 12 months post‐rupture. AT SWV was imaged from distal, middle, and proximal locations along the length of the tendon (ATdist, ATmid, ATprox). MVT was tested at 6‐ and 12 months. Achilles tendon total rupture score (ATRS) was inquired at 12 months. Pearson's correlation was used to assess the relationship between limb asymmetry (LSI, %) of ATmid SWV and the functional and structural outcomes. Linear mixed model was used to assess the effect of time, limb condition and imaging location on AT SWV. AT SWV LSI correlated with MVT LSI at 6‐ and 12 months after rupture (*r* = 0.500, *p* = 0.011; *r* = 0.653, *p* < 0.001, respectively). By 6 months, the injured tendon reached SWV level of the uninjured limb and at 12 months, ATprox SWV of the injured side was higher than the uninjured (mean difference 2.4 m × s⁻¹ (95% CI 0.9–3.7), *p* = 0.007. At 12 months, median ATRS was 91 points. Increase in SWV of the injured tendon may indicate an improvement in its material properties during recovery. This improvement may be associated with the ruptured tendon's ability to transfer loads and the muscle‐tendon units' capacity to generate force.

## Graphical abstract

During a 1‐year follow‐up after an acute Achilles tendon rupture, the injured tendon reached the shear wave velocity level of the uninjured limb by 6 months. At 6‐ and 12 months, the symmetry of tendon shear wave velocity correlated with the symmetry of ankle plantarflexion strength, but not with the number of daily steps nor the symmetry of tendon thickness. The median Achilles tendon total rupture score was 91 points at 12 months.

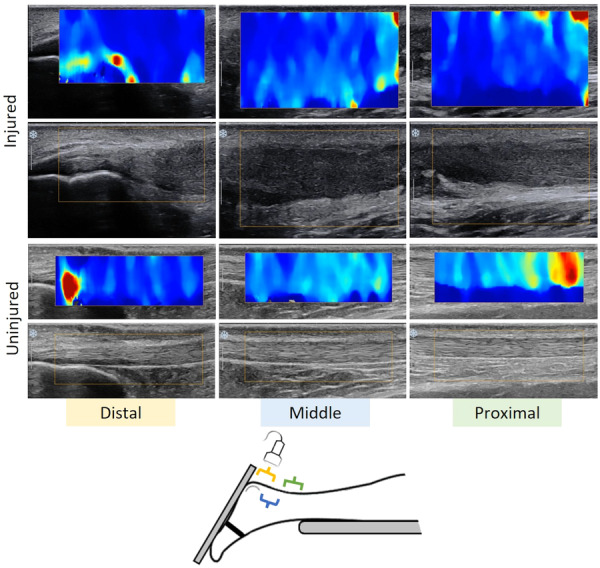

## 1 Introduction

Physiological healing of an Achilles tendon rupture (ATR) can take more than a year [1] and may result in persistent changes in the structural and mechanical properties of the tendon [2, 3]. Progressive tendon loading is key to recovery through mechanical and biochemical responses, as it facilitates rearrangement of collagen fibers and improves fiber cross‐linking and tendon architecture [4, 5, 6, 7]. These adaptations gradually increase the mechanical strength of the injured tendon [8], which is important for functional recovery. Ultrasound imaging has been used to quantify tendon mechanical properties after rupture [3, 9], and technological advances in imaging modalities, such as shear wave elastography [10], have provided new ways to monitor healing [11].

Shear wave elastography is a non‐invasive ultrasound technique used to assess the material properties of biological tissues [10] and has been suggested as a potential tool for monitoring musculoskeletal injuries [12, 13]. Acutely after ATR, the shear wave‐based stiffness of the Achilles tendon (AT) decreases [14, 15] and then gradually increases during healing [15, 16, 17, 18, 19]. Given that tendons adapt to diverse loading environments [20] and play an important role in the force transmission and force‐generating capacity of a muscle [21, 22], it is reasonable to hypothesize that there may be associations between elastic tendon properties and measures of daily function. Interestingly, a few studies have suggested that elastography measures of the AT post‐rupture might be associated with recovery of gait symmetry [18, 23], symmetry of isometric ankle plantarflexion strength [24], and patient‐reported tendon function [16, 17, 25]. For example, Zellers and colleagues [23] found a positive longitudinal association between the symmetry of AT dynamic shear modulus 1 month after rupture and the symmetry of stance phase parameters during walking at 6 months. However, the study did not find a relationship between the symmetry of AT dynamic shear modulus and the average number of daily steps at 3 months post‐rupture [23]. Further investigation is needed to determine how changes in AT SWV reflect various functional outcomes during tendon recovery over the course of longer follow‐ups following ATR.

The primary aim of this study was to characterize the longitudinal changes of AT SWV at 2‐, 6‐, and 12 months after rupture with the hypothesis that AT SWV of the injured limb would gradually increase to the level of the uninjured side. We further explored cross‐sectional correlations to assess the association between side‐to‐side symmetry of AT SWV and functional outcomes, which were quantified as the symmetry of isometric plantarflexion maximal voluntary torque (MVT), daily steps, and patient‐reported tendon function at each time point. We expected improved MVT symmetry [24], greater average daily step count [26, 27], and higher self‐reported tendon function [16, 17, 25] to be positively correlated with the symmetry of AT SWV. Additionally, the relationship between the recovery of tendon material properties and alterations in tendon geometry was explored by examining whether the symmetry of AT SWV was related to the symmetry of AT thickness.

## 2 Methods

Participants were recruited into a 1‐year follow‐up study “Non‐operative treatment of Achilles tendon rupture in Central Finland: a prospective cohort study” (trial registration: NCT03704532; level of evidence II) between 2018 and 2022. Recruitment was carried out at Hospital Nova of Central Finland at the time of diagnosis. Each participant presented with a unilateral ATR diagnosed within 14 days after acute injury according to the guidelines of the American Academy of Orthopaedic Surgeons [28]. Participants were treated with non‐operative care and early mobilization, as described previously [29]. All medical advice, as well as instructions regarding early mobilization and rehabilitation progression were given by the healthcare provider as part of usual care (Appendix A in Supporting material). Study inclusion criteria were a normal walking ability (> 100 m unaided) before ATR, permission from a physician to walk without orthosis before the first measurement, and a minimum age of 18 years. Exclusion criteria were a previous rupture in the contralateral limb, re‐rupture, avulsion fracture of the calcaneus, and systemic diseases that may associate with tendon health [30]. This study obtained approval from the Research Ethics Committee of the Central Finland Health Care District (2U/2018). All study procedures were performed in line with the Declaration of Helsinki. Participants read and signed informed consent before participation.

### 2.1 Data Collection

**Figure 1:**
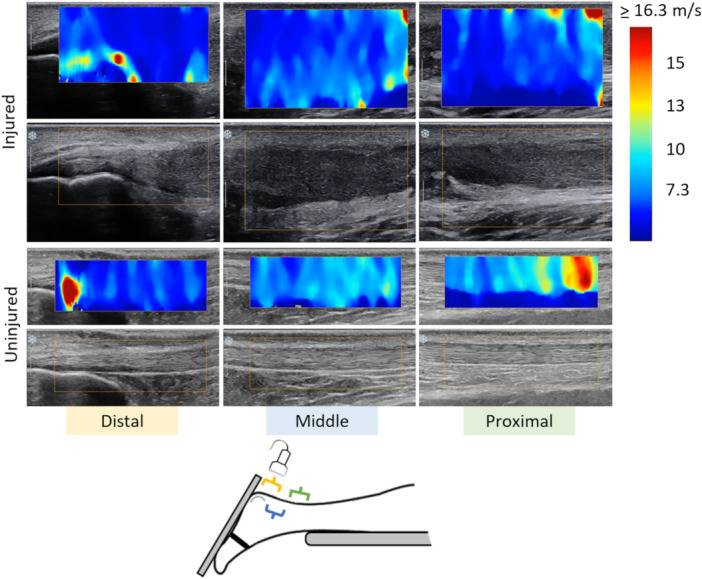
Shear wave elastography of the injured and uninjured limb from distal, middle, and proximal imaging locations over the tendon.

Data collection for the present study was carried out at the University of Jyväskylä. Shear wave elastography (Aixplorer Supersonic Imagine, v. 12.3.1 Aix‐en‐Provence, France) was used to record AT SWV [10] at rest. Shear wave imaging was performed according to methodology described elsewhere [31]. Briefly, a 38 mm linear transducer (2–10 MHz) was used to record AT SWV in longitudinal orientation from three overlapping tendon locations (ATdist, ATmid, ATprox) while participants were lying prone with both feet fixed at 25° ankle plantarflexion (Figure 1). The SWV speed range was set to 0–16.3 m × s⁻¹ and the imaging depth was adjusted according to the tendon thickness (range 2.5–3.0 cm). Limbs were imaged in random order; at each tendon location, a multi‐frame video with at least 3 elastograms was recorded. Intrarater reliability of SWV measurements (minimum of 24 h between trials) was tested for one rater with an additional pilot sample of healthy individuals (n = 12). Reliability was determined using the intra‐class correlation coefficient (ICC₃,₁), the coefficient of variation (CV), the standard error of measurement (SEM), and the minimum detectable change (MDC). Intrarater reliability of shear wave imaging between two trials yielded the following results: ICC 0.867 (0.523–0.968), SEM 0.7 m × s⁻¹, MDC 1.9 m × s⁻¹, and CV 6.6%.

AT thickness was measured in prone position with the ankle relaxed over the edge of the examination table. Imaging was performed longitudinally over the free tendon using a 36 mm linear transducer (Aloka Alpha‐10 system, Tokyo, Japan) [32].

Isometric plantarflexor MVT was tested using a custom‐made ankle dynamometer (Peltonen et al., 2010). Participants were seated with the hip flexed at 60° and the knee secured in extension. The foot was fixed at a neutral ankle angle (0°). The axis of the dynamometer was aligned according to the presumed center of rotation of the ankle. Before testing the MVT, participants performed ~2 min of submaximal contractions for conditioning. Ankle torque was recorded using Spike2 software (v. 6.17, Cambridge Electronic Design Limited, Cambridge, UK) and sampled at a frequency of 1 kHz using a 16‐bit analogue‐to‐digital converter (Power 1401, Cambridge Electronic Design, Cambridge, UK) connected to a computer. Participants were given a 2‐min rest between each MVT trial and a minimum of three trials were recorded.

At the end of each laboratory visit, each participant was given a tri‐axial accelerometer (ACC; RM‐42, UKK Institute, Tampere, Finland) attached to an adjustable waist belt, together with a daily diary and a prepaid envelope to return the device and completed diary by mail. The ACC stored acceleration at 100 Hz sampling rate with a 13‐bit A/D conversion of the ±16 g range. Participants were instructed to wear the ACC over the iliac crest on the right side during waking hours for seven consecutive days, except during water‐based activities. Participants were asked to write down the times they wore the ACC in the diaries.

Perception of tendon recovery was assessed using Achilles tendon total rupture score (ATRS) [33] at 12 months post‐rupture. The Finnish translation of ATRS is calculated between 0 and 100 points, with 100 points indicating no functional deficits.

Sample size was determined based on previous literature that reported changes in shear wave elastography findings after ATR [15, 16, 17, 18, 19] or assessed associations between elastographic and other objective outcomes after ATR [18, 34].

### 2.2 Data Processing

Shear wave elastography recordings were processed using a custom software (ElastoGUI, University of Nantes, France) developed for MATLAB (v. R2023b, MathWorks Inc, Natick, MA, USA) with semi‐automatic processing. Each pixel of the elastograms was converted to SWV based on the recorded color scale. Processed videos contained a minimum of three elastograms (range 3–10) and an average of all frames was used for later analysis. For each SWV recording, the area of analysis was adjusted to cover the largest possible area within the tendon borders while avoiding artifacts. Acceptable saturation and void levels of < 3% and < 0.5% were used. In results, AT SWE measurements are reported as mean SWV (m × s⁻¹). All SWE data are provided in both in SWV and kPa in the Supporting Material (Appendix B) to facilitate scientific data comparison.

Mean ± standard deviation (SD) values of the analyzed areas were: ATdist 0.5 ± 0.1 cm [2]; ATmid 1.0 ± 0.3 cm^2^; ATprox 0.9 ± 0.3 cm^2^ in the uninjured and ATdist 0.9 ± 0.4 cm^2^; ATmid 2.6 ± 0.7 cm^2^; ATprox 2.6 ± 0.7 cm^2^ in the injured limb, respectively. Saturation was present in 259 out of 462 analyzed recordings (average saturation % in different locations: ATdist 0.2%; ATmid 0.3%; ATprox 0.4% in the uninjured and ATdist 0.2%; ATmid 0.3%; ATprox 0.5% in the injured limb, respectively).

Tendon thickness was determined using ImageJ (1.44b, National Institutes of Health) 2 cm above the proximal head of the calcaneus. The average values from two images were used for further analysis.

Limb asymmetry index (LSI) [35] was calculated as a percentage (%) difference between uninjured and injured limb: $\frac{\mathrm{Injured}-\mathrm{Uninjured}}{\mathrm{Uninjured}}\times 100$.

Days with at least 10 h of ACC wear time for at least 4 consecutive days [36] were considered eligible for further analysis. The daily step counts were analyzed using commercial ActiLife Software (v. 6.13.4). Non‐wear time was determined as any ≥ 60‐min epoch where 1‐min count values were continuously zero using custom written scripts. This methodology followed well the reported wear times in participants' diaries. Total steps during each day were averaged for each participant. The average wear time across all time points was 14.6 ± 1.3 h per day and 6.8 ± 0.72 days per week.

### 2.3 Statistical Analysis

Statistical analyses were performed with JASP (v. 0.16.4, Amsterdam, Netherlands) and partly illustrated with Jamovi (v. 2.2.5, Sydney, Australia). Continuous variables are presented as mean ± SD or median with interquartile range (IQR). Statistical significance was defined as *p* < 0.05. Normal distribution was evaluated from the skewness and kurtosis of the data. Linear mixed model was used to assess the effect of time and limb condition on AT SWV in the three AT imaging locations. A linear mixed model was built with AT SWV as the dependent variable and limb condition (injured vs uninjured), time (2, 6 and 12 months) and imaging location (distal, mid and proximal) as fixed factors. Participant was used as a random factor. Linear mixed model was also used to assess the change in MVT, daily step count, and AT thickness over time, with time and limb condition (only time for step count) as fixed factors and participant ID as a random factor. Estimated marginal means contrasts were performed for significant models with Bonferroni correction. Models were fitted using restricted maximum likelihood as the estimation method. Model terms were tested using Satterthwaite's type III sum of squares method. Results are presented as estimated means and 95% confidence intervals (CI). Pearson's correlation coefficient was used to calculate partial correlations controlled for age between ATmid SWV LSI and MVT LSI, AT thickness LSI, average daily step count, and ATRS score. We chose to use LSI calculations for the bilaterally measured outcomes in the correlation analysis because it individually reflects the recovery of the injured limb in relation to the uninjured side. ATmid imaging location was chosen for the correlation analysis to simplify the results of this research and because it corresponds to the free tendon area in most individuals [37].

## 3 Results

Twenty‐seven individuals (19 males, 8 females; mean ± SD age 41.5 ± 9.8 years, height 176.4 ± 8.7 cm, body mass 84.5 ± 16.5 kg, body mass index 27.0 ± 3.9 kg/m^2^) with unilateral ATR participated in this study with measurements performed (mean ± SD) at 2.2 ± 0.2, 6.7 ± 1.1, and 12.6 ± 0.7 months after ATR.

### 3.1 Missing Data

**Figure 2:**
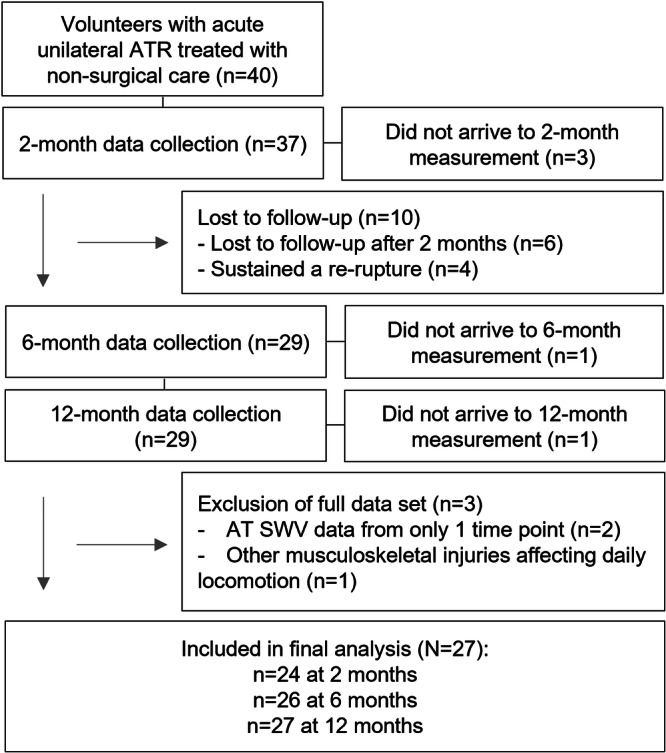
Flow diagram showing the participation and dropouts during the data collection at 2‐, 6‐, and 12‐month measurements after Achilles tendon rupture and the subsequent inclusion and exclusion of data in the final analysis.

Three participants were unable to attend measurements at 2‐month and one participant at 6‐month time points due to personal reasons (Figure 2). Three participants had no valid ACC data at 2 months, four participants at 6 months, and one participant at 12‐month time point due to either un‐returned ACC or failed data collection. Tendon thickness images were missing for one participant at 2‐month time point, and ATRS questionnaire was not completed for 3 participants at 12 months.

### 3.2 Shear Wave Velocity Across Time Points

Linear mixed model showed a significant effect of time, limb condition and imaging location on AT SWV (time F (2–263.49) = 37.7; limb condition F (1–259.86) = 18.1; imaging location F (2–259.86) = 47.5; *p* < 0.001 for all). A significant interaction was found between time and limb condition (F (2–259.86) = 20.4, *p* < 0.001) and between limb condition and imaging location (F (2–259.86) = 4.1, *p* = 0.018).

**Figure 3:**
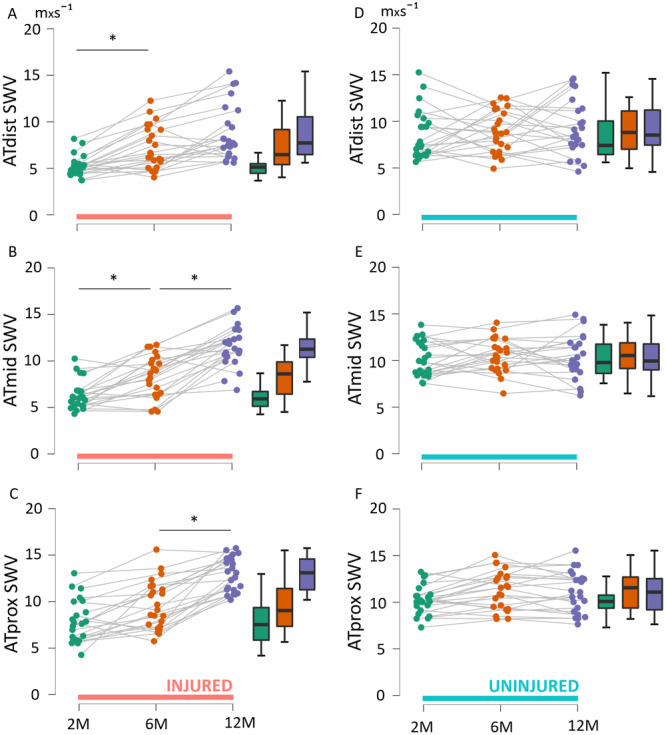
(A–C) Shear wave velocity of the Achilles tendon in the injured limb across the three time points in distal (A), middle (B) and proximal (C) imaging locations; (D–F) shear wave velocity of the uninjured limb across the three time points in distal (D), middle (E) and proximal (F) imaging locations with * indicating statistically significant difference between time points.

In the injured tendon, ATdist SWV increased from 2 to 6 months with a mean difference of 2.2 m × s⁻¹ (95% CI: 0.743–3.648, *p* = 0.021), but no statistical difference was found between 6 and 12‐month time points (Figure 3). The ATmid SWV of the injured limb showed an increase from 2 to 6 months and from 6 to 12 months (2.4 m × s⁻¹, 95% CI: 0.932–3.835, *p* = 0.009; 3.0 m × s⁻¹, 95% CI: 1.519–4.379, *p* < 0.001, respectively). For ATprox, an increase in SWV was only found between the 6‐ and 12‐month time points (3.3 m × s⁻¹, 95% CI: 1.892–4.751, *p* < 0.001) in the injured limb. No changes in AT SWV were observed on the uninjured side during follow‐up.

**Figure 4:**
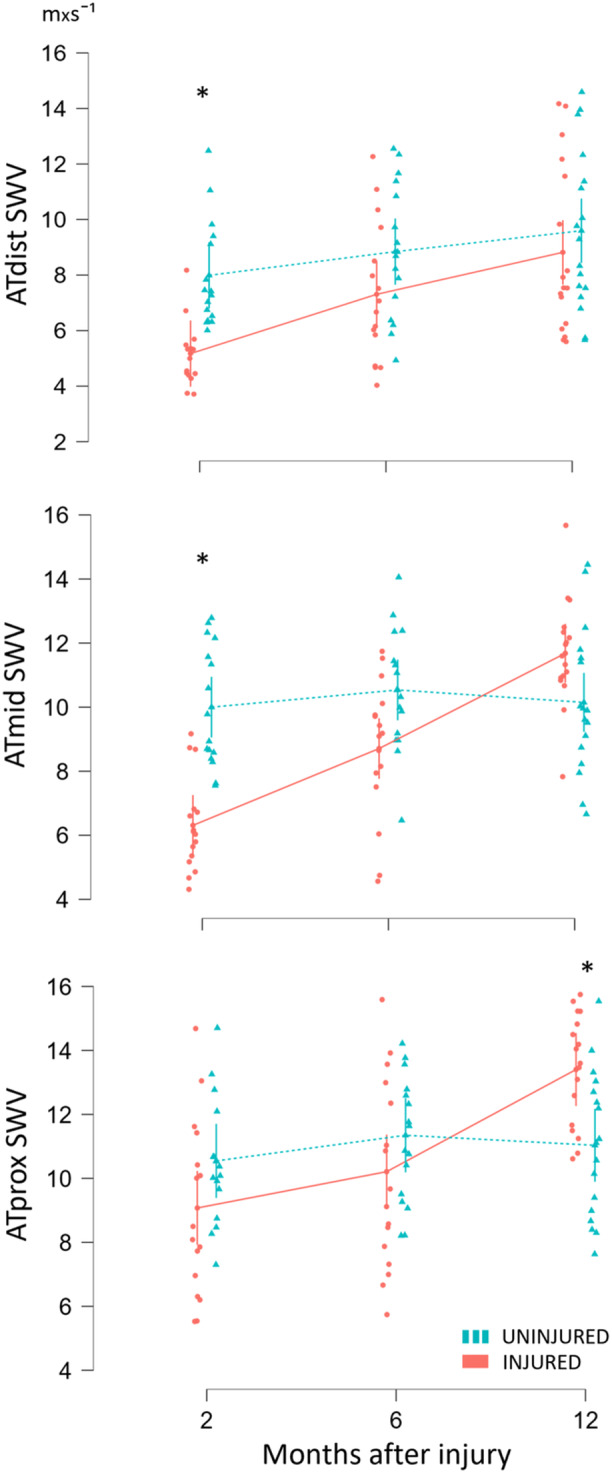
Difference in shear wave velocity between limbs across three time points for each imaging location (distal in upper panel, middle in mid panel and proximal in lower panel) with * indicating statistically significant difference between limbs.

A lower SWV was found for ATdist and ATmid in the injured limb at 2 months compared to the uninjured side (−2.8 m × s⁻¹, 95% CI: −4.3 to −1.4, *p* < 0.001; −3.7 m × s⁻¹, 95% CI −5.1 to −2.2, *p* < 0.001; respectively) (Figure 4). No differences were observed between limbs at any of the imaging locations at 6 months. At 12 months, ATprox of the injured tendon showed higher SWV compared to the uninjured side (2.4 m × s⁻¹, 95% CI: 0.9–3.8, *p* = 0.007).

### 3.3 Correlation Analysis

**Figure 5:**
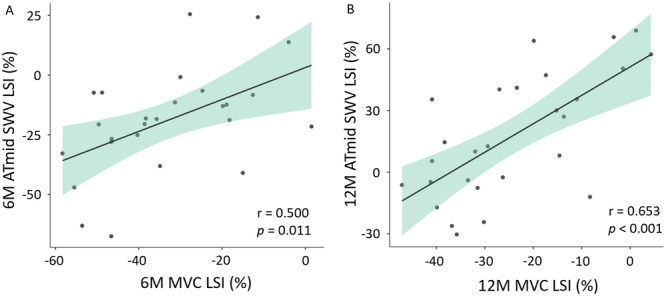
Partial correlations between AT shear wave velocity LSI and MVT LSI at 6‐ (A) and 12 months (B) after rupture. ATmid = middle Achilles tendon imaging location, LSI = limb asymmetry index, MVT = Maximum voluntary torque, SWV = Shear wave velocity.

The middle AT imaging location SWV LSI was positively associated with MVT LSI at both 6‐ and 12 months after rupture (Figure 5). We found no statistical correlation between ATmid SWV LSI and AT thickness LSI or daily step count at any time point, nor with ATRS score at 12 months. Scatter plots for the non‐significant correlations can be found in the Supporting material (Appendix C in Supporting material).

### 3.4 Functional and Structural Outcomes

Between 6 and 12 months, the MVT improved in both the injured and uninjured limbs (mean difference 37.1 Nm, 95% CI 25.1–49.1, *p* < 0.001 for the injured limb; 19.3 Nm, 95% CI: 7.3–31.2, *p* = 0.009 for the uninjured limb). The uninjured limb showed higher ankle plantarflexion strength than the injured limb at both time points (79.7 Nm, 95% CI: 61.8–97.6, *p* < 0.001 at 6 months; 61.9 Nm, 95% CI 44.1–79.6, *p* < 0.001 at 12 months). Average daily steps increased from 2‐ to 6 months (mean difference 2257 steps, 95% CI: 1251–3264, *p* < 0.001). Throughout the follow‐up, the thickness of the injured tendon first increased from 2‐ to 6 months (0.22 cm, 95% CI: 0.18–0.27, *p* < 0.001) and then decreased from 6‐ to 12 months (−0.18 cm, 95% CI: −0.29 to −0.14, *p* < 0.001) with no statistical changes on the uninjured side. At each time point, the injured tendon was thicker compared to the uninjured side (0.47 cm, 95% CI: 0.39–0.54, *p* < 0.001 at 2 months; 0.71 cm, 95% CI 0.64–0.79, *p* < 0.001 at 6 months; 0.530 cm, 95% CI: 0.45–0.60, *p* < 0.001 at 12 months). The absolute values of the functional and structural outcomes are shown in Table 1. At 12 months, the median (IQR) ATRS score was 91 (16) points.

**Table 1:** Descriptive statistics of maximal voluntary contraction, Achilles tendon thickness and daily step count during the follow‐up.

	2 months (Mean ± SD)		6 months (Mean ± SD)		12 months (Mean ± SD)	
	Injured limb	Uninjured limb	Injured limb	Uninjured limb	Injured limb	Uninjured limb
MVT (Nm)	na	na	157.3 ± 66.5	235.1 ± 68.6	193.5 ± 74.2	255.4 ± 77.4
AT thickness (cm)	0.97 ± 0.19	0.49 ± 0.08	1.19 ± 0.20	0.47 ± 0.08	1.00 ± 0.21	0.47 ± 0.07
Daily step count	5551 ± 1998		6916 ± 2758		7188 ± 2455	

Abbreviations: AT, Achilles tendon; MVT, maximum voluntary torque; na, not assessed; Nm, Newton meter; SD, standard deviation.

## 4 Discussion

The primary aim of this study was to explore the relationship between AT SWV symmetry and different functional recovery outcomes at 2‐, 6‐, and 12 months after ATR. The results showed that improved symmetry of AT SWV was associated with improved ankle plantarflexion MVT at 6‐ and 12 months after rupture, without statistical associations between AT SWV and average daily step count nor ATRS score. Additionally, the results showed that the SWV of the injured tendon gradually increased, reaching the SWV level of the uninjured side by 6 months. At 12 months, the injured tendon had a higher SWV than the uninjured side at the proximal imaging location. The findings of this study align with previous research [18, 23, 24] suggesting that AT SWV may correspond to patient function within the first year of injury and could potentially identify individuals with slow or poor recovery.

### 4.1 Association Between AT SWV and Patient Function

Along with our previous longitudinal findings [24], this study is the first to show a relationship between AT SWV and ankle plantarflexion strength deficit during recovery from ATR. Our recent report showed that AT SWV LSI measured 2 months after rupture was associated with MVT LSI 6‐ and 12 months after ATR [24]. In addition, other studies have found associations between elastography findings and objectively measured patient function following rupture [18, 23]. Using continuous shear wave elastography, Zellers and colleagues [23] reported a relationship between tendon shear modulus at 1 month and gait symmetry at 6 months post‐rupture, while Laurent et al. [18] showed that improved AT SWV symmetry was associated with more symmetrical foot plantar pressure parameters measured with in‐shoe insoles during walking within 3 months of injury. Given that the AT plays a crucial role in force transmission during locomotion and influences the force‐generation capabilities of the plantarflexors, the observed correlation between AT SWV LSI and MVT LSI may indicate that participants with greater symmetry have improved tendon function and therefore better overall recovery within the first year after ATR.

We did not find a correlation between AT SWV LSI and daily step count, which aligns with previous findings showing that the average daily step count assessed 3 months post‐rupture was not associated with AT dynamic shear modulus symmetry [23]. Previously, device‐monitored step counts have only been reported from the first 2 weeks after ATR [38, 39], and to date, longer follow‐ups have been lacking. In light of the limited evidence available, we aimed to explore whether the number of daily steps would be associated with the material properties of the healing tendon through repetitive loading [6, 26, 27, 40]. Walking at an average speed of 1.5–1.6 m × s⁻¹ results in peak AT loads of 3–4 times body weight [26, 40], a level that exceeds the loads borne during some commonly used rehabilitative exercises early after injury [26, 27]. However, ACC‐derived step counts only assess the frequency of loading and do not reflect altered gait strategies in the injured limb. Therefore, closer investigation of the quality and magnitude of daily tendon loads may be necessary to detect meaningful differences.

No correlation was observed between AT SWV LSI and 12‐month ATRS score, which is consistent with previous studies [41, 42]. This was expected as the ATRS assesses an individual's perception of tendon recovery and does not explain the condition and quality of tendon material properties. Patient‐reported outcomes and objective measures do not necessarily agree after ATR as they measure different constructs, however, some studies have observed a relationship between AT elastography findings and clinical scores [16, 17, 18, 25]. Overall, the average ATRS score in this study suggests that the majority of participants had little or no perceived deficits at 12 months after non‐operatively treated rupture.

### 4.2 Changes in AT SWV After Rupture

The gradual increase of SWV in the injured tendon was consistent with previous follow‐up studies using shear wave elastography after ATR [15, 16, 17, 19]. We found that the injured tendon regained SWV level of the uninjured side by the 6 months' time point. Previous studies showed no difference between limbs in ultrasound elastography variables already 3 months after injury [15, 18, 19], whereas some reported lower values on the injured side at 1 year [17] and at least 2 years [25, 41] after rupture. In general, direct comparison of shear wave elastography results between studies is limited by differences in study protocols, including participant position [43, 44, 45] and imaging location over the tendon [13, 31, 46]. Despite these differences, it appears that there may be inter‐individual variation in the recovery of tendon material properties when comparing to the uninjured side, as similarly inconsistent findings have been reported in studies assessing force‐elongation curves via dynamometry and B‐mode ultrasound [3, 32, 47]. Since mechanical loading is essential for tendon remodeling [4, 6, 7], the observed differences in the recovery of tendon material properties may arise from different loading patterns between individuals. More detailed monitoring of patient engagement in rehabilitation exercises, the intensity and type of daily physical activity, and quantitative measurements of tendon loads during recovery could improve understanding of the influence of loading on the material properties of the injured tendon.

The ATprox SWV of the injured limb exceeded that of the uninjured by 12 months, in agreement with recent findings by Yoshida and colleagues [19]. Acutely after rupture, the shear wave‐based stiffness is markedly reduced due to tendon fiber separation [14, 15, 19], and the subsequent increase in SWV is considered a sign of improvement in tendon resilience and load‐bearing capacity as physiological healing progresses [15]. Following rupture, tendon elongation and subsequent shortening of triceps surae muscle fascicles require the tendon to re‐adjust its mechanical properties by increasing stiffness to improve plantarflexion force production [48]. In the present study, the correlation between AT SWV LSI and MVT LSI suggests that SWV may reflect the regain of tendon tensile strength that is needed for efficient force transmission through the injured AT. Therefore, higher SWV of the injured tendon could indicate successful recovery, and conversely a lower SWV of the injured side compared to the uninjured side may suggest ongoing or delayed tendon healing. However, it is unclear how tendon properties measured at low forces manifest during dynamic movements involving high forces [9]. Therefore, the findings of this study warrant careful interpretation.

### 4.3 Association Between AT SWV and Tendon Thickness

The structure of the AT has been shown to influence shear wave propagation [49] and SWV can decrease with increasing imaging depth [46]. In this study, the results did not show an association between AT SWV LSI and tendon thickness LSI, which aligns with prior studies in both healthy individuals [50] and patients following surgical repair of ATR [42]. A previous study observed a weak correlation between the AT thickness and SWV, with decreasing SWV along with decreasing tendon thickness [46]. As we were interested in the symmetry between the injured and uninjured legs, the relationship between AT SWV and AT thickness was assessed using LSI values. Furthermore, during data processing, AT thickness was measured from a set location 2 cm proximal to the calcaneus, whereas SWV was averaged over an area of ~1.0 cm^2^ in the uninjured and ~2.6 cm^2^ in the injured tendon.

### 4.4 Limitations

Similarly to Lin et al. [51], a single constant artifact in SWV color maps was identified in the tendon region which appeared as an area with lower estimated SWV values (Appendix D in Supporting material). The artifact was removed from the analysis by carefully outlining it from the region of interest on the elastogram. Therefore, the artifact should not have influenced the results obtained. Furthermore, the rehabilitation protocol was not controlled or systematically recorded. Participants received the same instructions for early mobilization, weight bearing, and progressive rehabilitation but they may have sought additional support for their rehabilitation and variability in loading regimens may have influenced tendon recovery. Additionally, we had no information on the initial gap size between the tendon stumps, which may have led to differences in the recovery of tendon material properties between individuals [52]. It should also be noted that AT SWV and plantarflexor muscle MVT were tested at different ankle angles, which may have influenced the results. This study was part of a larger research project, in which the neutral ankle angle was chosen for testing MVT because it is representative of lower limb function in the upright position during various functional tasks. Tendon SWV imaging was performed in a plantarflexed ankle position because the inherent technical limitations of ultrasound shear wave elastography could have resulted in data loss due to excessive pixel saturation when imaging tendon beyond its slack length. In addition, due to time constraints, we could not perform SWE imaging reliability assessment within the laboratory visits and therefore, an additional pilot sample of healthy individuals was used. As only reliability data from healthy tendons was available, it is unclear whether changes in ruptured AT SWV exceed the measurement error. Furthermore, the inclusion criterion of being able to walk unaided at 2 months post‐rupture may have biased the cohort towards individuals with a good recovery by excluding those with slower healing. As this study was part of a larger cohort study testing various recovery parameters, clearance from medical practitioners regarding safe ambulation was required to ensure the safe inclusion of participants. Lastly, the study included a limited number of females, however, the sex distribution was typical for an ATR cohort [53].

## 5 Conclusions

The SWV of the injured limb gradually increased during the 1‐year follow‐up. At 12 months, the SWV was higher in the injured limb compared to the uninjured, which may reflect adaptations at the tendon cellular level during rehabilitation. The novel observation that the symmetry of AT SWV is correlated with the symmetry of isometric plantarflexor muscle MVT suggests that shear wave elastography may be a potential tool to identify patients with prolonged loss of plantarflexor muscle strength at a given time point of recovery. Due to its non‐invasive nature and ease of use, shear wave elastography could also be a valuable tool to indicate healing measured in resting conditions.

## Author Contributions

Taija Finni and Neil J. Cronin conceived and designed the research. Taija Finni and Maria Sukanen obtained funding for the study. Taija Finni and Arto J. Hautala were responsible of supervision. Taija Finni, Ra'ad M. Khair, Maria Sukanen, Iida Laatikainen‐Raussi, Aleksi Reito, and Ville Ponkilainen contributed to the data collection. Maria Sukanen and Iida Laatikainen‐Raussi were responsible for data analysis. Maria Sukanen conducted statistical analysis and drafted the manuscript. All authors contributed to the writing of the final manuscript and critically revised the report for important intellectual content. All authors approved the final manuscript.

## Ethics Statement

Ethical approval was granted by the Research Ethics Committee of the Central Finland Health Care District (2U/2018). All procedures performed in this study were in accordance with international ethical standards.

## Conflicts of Interest

The authors declare no conflicts of interest.

## Supporting information

Supporting material 1.

Supporting material 2.

Supporting material 3.

Supporting material 4.

## Data Availability

The data that support the findings of this study are available from the corresponding author on a reasonable request.
