# Supplementary material for: Achilles Tendon Shear Wave Velocity Within a 1‐Year Follow‐Up After Non‐Operatively Treated Rupture
Source: J Orthop Res. 2026 Apr 1;44(4):e70201. doi: 10.1002/jor.70201 (PMC13040326; doi:10.1002/jor.70201)
Supplement: Supplementary file 1 — Supporting material 1. [file JOR-44-0-s001.docx]

| **Appendix A**. Non-operative care with early mobilization and progressive rehabilitation after Achilles tendon rupture. | |
| --- | --- |
| Week 0-2 | Full equinus ankle cast. |
| Week 2-4 | Functional walking cast with 20° equinus.  Active plantarflexion exercises without load.  Instructions to proceed to full weightbearing by week 4. |
| Week 4-8 | Functional walking orthosis with 1 cm heel wedge.  Removal of the heel wedge from the orthosis at week 6. |
| Week 8 | Medical practitioners’ clearance to walk unaided.  Removal of orthosis.  Instructions to use heel wedge in a shoe for weeks 8-12.  Instructions for independent progressive rehabilitation from a physiotherapist. |
| Week 9-26 | Progressive exercises 2-3 times a week improving the symmetry of ankle mobility, strength, and balance.  Walking, cycling, water activities, and overall strength training.  Gradual return to jogging. |
| Week 26-52 | Maximal plantarflexion efforts.  Return to team and racquet sports. |

**Supplementary material 1** in the manuscript Sukanen et al. Achilles tendon shear wave velocity within a 1-year follow-up after non-operatively treated rupture.
