# Supplementary material for: Achilles Tendon Shear Wave Velocity Within a 1‐Year Follow‐Up After Non‐Operatively Treated Rupture
Source: J Orthop Res. 2026 Apr 1;44(4):e70201. doi: 10.1002/jor.70201 (PMC13040326; doi:10.1002/jor.70201)
Supplement: Supplementary file 2 — Supporting material 2. [file JOR-44-0-s003.docx]

**Supplementary material 2** in the manuscript Sukanen et al. Achilles tendon shear wave velocity within a 1-year follow-up after non-operatively treated rupture.

| **Appendix B.** Descriptive statistics of regional Achilles tendon shear wave velocity (SWV) and shear modulus (kPa). | | | | | |
| --- | --- | --- | --- | --- | --- |
|  | Imaging location | Injured limb (mean±SD) | | Uninjured limb (mean±SD) | |
|  |  | SWV | kPa | SWV | kPa |
| 2M | ATdist | 5.3±1.1 | 30.0±14.7 | 8.7±2.6 | 83.7±52.7 |
|  | ATmid | 6.3±1.5 | 44.0±24.5 | 10.2±1.9 | 111.2±40.9 |
|  | ATprox | 8.1±2.7 | 77.2±50.8 | 10.4±1.8 | 115.4±40.3 |
| 6M | ATdist | 7.2±2.2 | 60.0±38.7 | 8.9±2.3 | 86.9±44.5 |
|  | ATmid | 8.4±2.2 | 81.8±39.0 | 10.5±1.8 | 119.1±39.5 |
|  | ATprox | 9.8±2.6 | 109.3±55.3 | 11.2±2.1 | 113.8±48.5 |
| 12M | ATdist | 8.9±2.9 | 92.0±59.8 | 9.0±2.9 | 92.6±58.4 |
|  | ATmid | 11.6±2.1 | 148.5±46.7 | 10.3±2.3 | 115.4±50.4 |
|  | ATprox | 13.1±1.8 | 182.2±43.8 | 11.1±2.0 | 131.4±47.4 |
| SD=Standard deviation; AT=Achilles tendon; ATdist, ATmid, ATprox=Distal, middle, and proximal imaging location of the AT; 2M, 6M, 12M=Measurements at 2-, 6-, and 12 months after AT rupture | | | | | |

The reported shear modulus (μ) is calculated from shear wave velocity (*μ* = *ρV*^2^ (*ρ* = muscle mass density 1000 kg m^3^) (Bercoff et al., 2004).

Bercoff J, Tanter M, Fink M. 2004. Supersonic shear imaging: a new technique for soft tissue elasticity mapping. *IEEE Trans Ultrason Ferroelectr Freq Control*: 51(4):396-409. doi:10.1109/tuffc.2004.1295425
