# Supplementary material for: Achilles Tendon Shear Wave Velocity Within a 1‐Year Follow‐Up After Non‐Operatively Treated Rupture
Source: J Orthop Res. 2026 Apr 1;44(4):e70201. doi: 10.1002/jor.70201 (PMC13040326; doi:10.1002/jor.70201)

**Supplementary material 3** in the manuscript Sukanen et al. Achilles tendon shear wave velocity within a 1-year follow-up after non-operatively treated rupture.

**
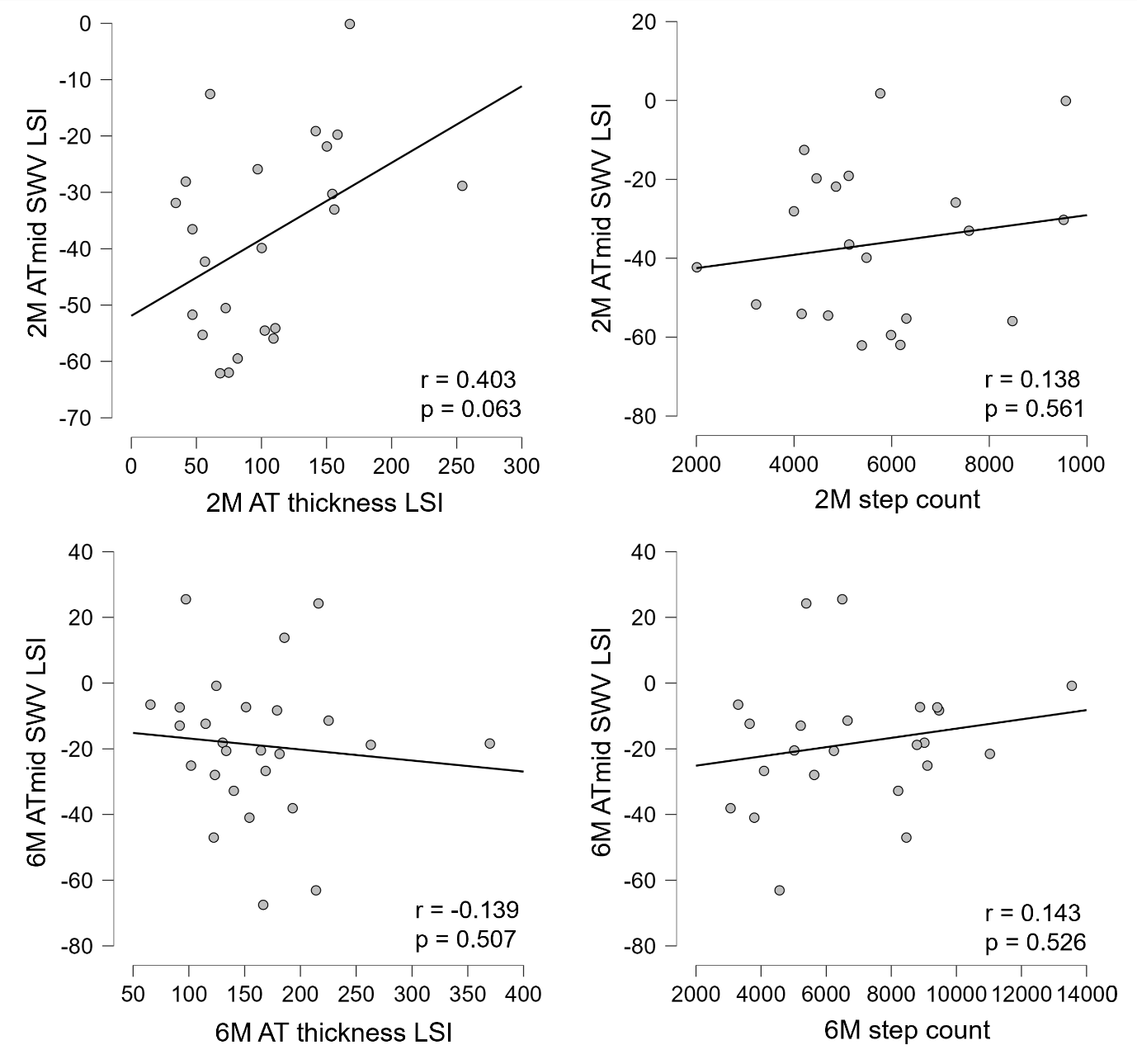
Appendix C.** Scatter plots showing cross-sectional non-significant correlations between the limb asymmetry index (LSI) of Achilles tendon (AT) shear wave velocity (SWV) at the mid-free tendon region and both AT thickness LSI and average daily step count at 2-, 6-, and 12 months after AT rupture.


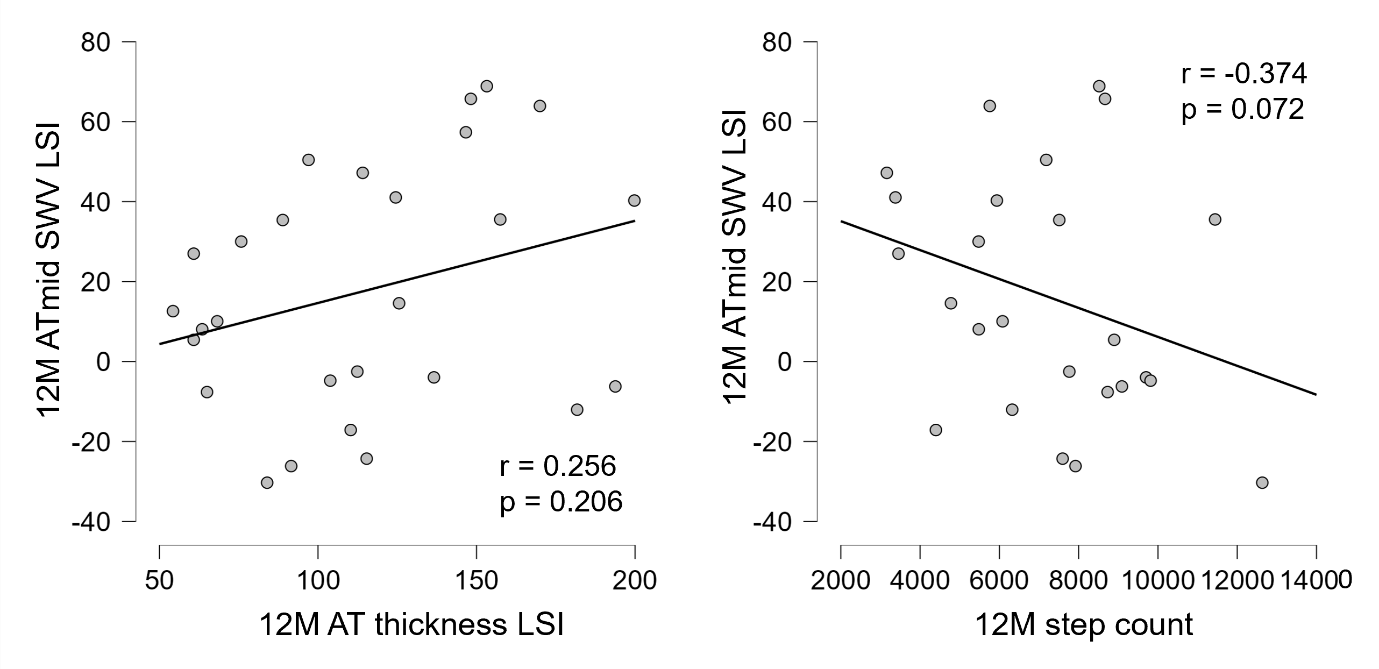

Supplement: Supplementary file 3 — Supporting material 3. [file JOR-44-0-s002.docx]
