# Supplementary material for: Achilles Tendon Shear Wave Velocity Within a 1‐Year Follow‐Up After Non‐Operatively Treated Rupture
Source: J Orthop Res. 2026 Apr 1;44(4):e70201. doi: 10.1002/jor.70201 (PMC13040326; doi:10.1002/jor.70201)
Supplement: Supplementary file 4 — Supporting material 4. [file JOR-44-0-s004.docx]

**Supplementary material 4** in the manuscript Sukanen et al. Achilles tendon shear wave velocity within a 1-year follow-up after non-operatively treated rupture.


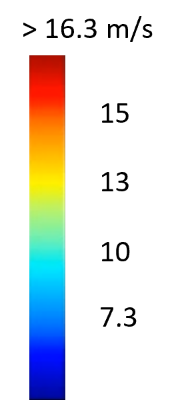

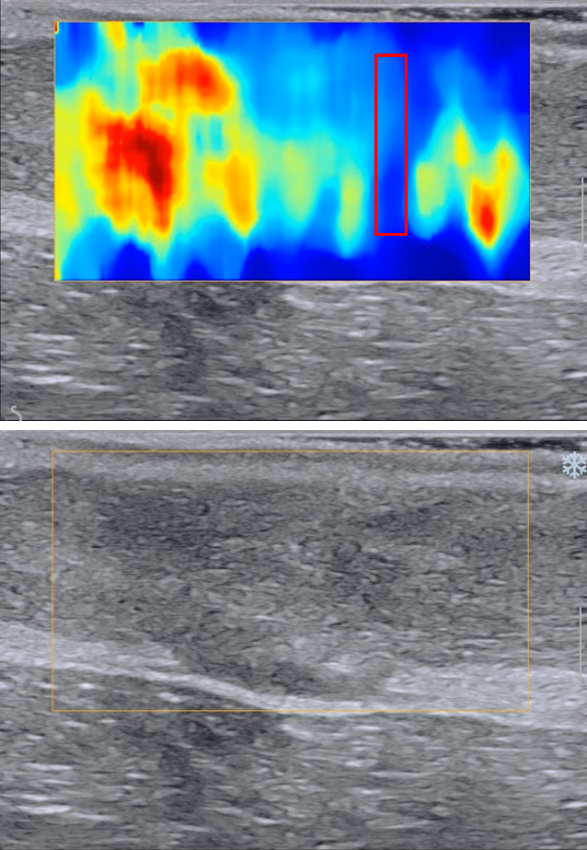

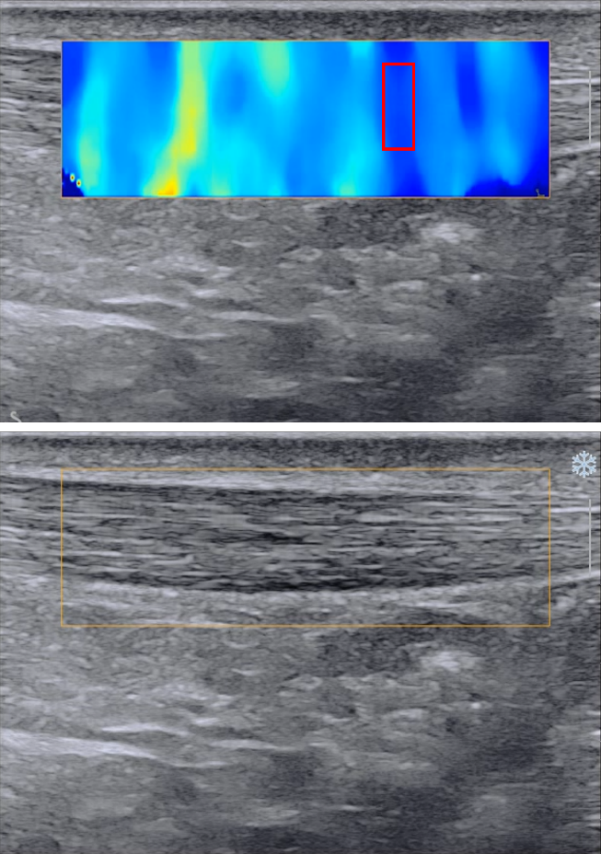

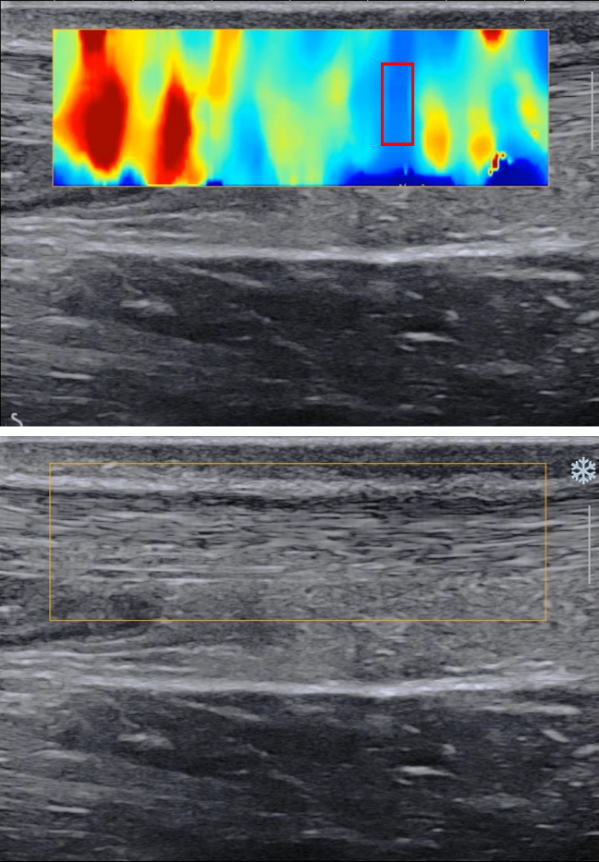
**Appendix D.** Example pictures showing an artefact (red rectangles) that was observed within the elastogram from shear wave elastography imaging.
